# The effect of purified *Quercus cortex* extract on biochemical parameters of organism and productivity of healthy broiler chickens

**DOI:** 10.14202/vetworld.2018.235-239

**Published:** 2018-02-23

**Authors:** Galimzhan Kalihanovich Duskaev, Nadezhda Mihajlovna Kazachkova, Alexander Sergeevich Ushakov, Baer Serekpaevich Nurzhanov, Albert Farhitdinovich Rysaev

**Affiliations:** 1 Department for Feeding Agricultural Animals and Fodder Technology, All-Russian Research Institute of Beef Cattle Breeding, Federal Agency of Scientific Organizations, Orenburg, 460000, Russia; 2 Department of Food Biotechnology, Orenburg State University, Orenburg, 460018, Russia; 3 Department for Feeding Poultry, All-Russia Research and Technological Institute of Poultry, Sergiev Posad, 141311, Russia

**Keywords:** blood, broilers, growth, iron, *Quercus cortex*

## Abstract

**Aim:**

Modern methods of producing poultry meat without the use of antibiotics are known, and it is possible to achieve the desired conditions, including the use of herbal preparations. In addition, it is known that metabolites of medicinal plants are inhibitors of the quorum sensing system in bacteria. The aim of the present study was to determine the effect of *Quercus cortex* extract in a reduced dose on the productivity and body state of healthy chicken broilers.

**Materials and Methods:**

For the experiment, 120 heads of 7-day-old healthy broiler chickens were selected, and they were divided into four groups (n=30, 3 replicates of 10 birds in each group) by the analog method. The composition of diets of the experimental Groups I and II additionally included *Q. cortex* extract and Groups II and III included an enzyme preparation containing glucoamylase and concomitant cellulolytic enzymes. The following methods of study were used; gas chromatography–mass spectrometry, mass spectrometry and atomic emission spectrometry, and hematological analysis.

**Results:**

It was established that the increase in live weight of broiler chickens in experimental groups exceeded the analogous indicator in the control group by 3.1-16.6%, and feed intake within the entire experimental period increased by 2.6-15.4%, against a background of decreasing feed consumption for a weight gain of 1 kg of live weight (by 3.7-9.2%). There was an increase in iron concentration in blood of broiler chickens in Groups I and II (7.8-11.8%), in liver (23.7-92.4%, p≤0.05), and in spleen (53.9-77.7%, р≤0.05) against the background of a decrease in muscle tissue. A decreased content of monocytes and granulocytes was found, especially in experimental Group I.

**Conclusion:**

In the experiment, it was shown for the first time that the inclusion of *Q. cortex* extract in an enzyme-containing diet (anti-quarantine substances) was found to increase the productivity of poultry.

## Introduction

The effect of antimicrobial growth promoters (fodder antibiotics in livestock production) on the development of resistant bacteria and, as a consequence, the ban on their use in the European Union has led to an intensive search for effective alternative growth promoters. Modern methods of poultry meat production without the use of antibiotics are already known, and it is possible to achieve the desired conditions [1], including using herbal preparations, such as diallyl disulfide of garlic [2] and substances containing tannins [3,4]. Plant extracts, also known as phytobiotics, are used in animal feeding, in particular as antimicrobial, anti-inflammatory, antioxidant, and antiparasitic agents [5-7].

Many plants are multifunctional, and biologically active substances isolated from them have useful properties. Biologically active plant components are mainly secondary metabolites (phenolic compounds, aldehydes, ketones, ethers, and lactones) [8]. In addition, medicinal plants are inhibitors of the quorum sensing (QS) system in bacteria [9]. Such inhibitors are found in the *Quercus cortex* extract [10]. At the same time, it is known that exogenous enzymes not only increase the productivity of farm animals but also contribute to the development of bacterial flora in the gastrointestinal tract and thus indirectly affect bacteria population [11,12].

The research objective was to study the effect of the purified *Q. cortex* extract on biochemical parameters of the organism and productivity of healthy broiler chickens, including against the background of a diet containing enzymatic preparations.

## Materials and Methods

### Ethical approval

Poultry maintenance and procedures during the experiments met the requirements of the instructions and recommendations of Russian regulations (Order of the Ministry of Health of the USSR No. 755 of 12.08.1977) and “The Guide for Care and Use of Laboratory Animals (National Academy Press, Washington, D.C., 1996).”

### Study area

The study was carried out in the conditions of the Common Use Center with scientific equipment of the All-Russian Research Institute of Beef Cattle Breeding on “Smena-8” broiler chickens.

### Experimental design

For the experiment, 120 heads of 7-day-old chicken broilers were selected. Animals were divided into four groups (n=30, 3 replicates of 10 birds in each group), and a pair-analog method was used. During the experiment, all birds were kept in the same conditions. Basic diets (BD) (Table-1) for the experimental birds were prepared during the study, considering the recommendations of the All-Russian Research and Technological Poultry Institute [13], as follows. Control group – BD; I group – BD + *Q. corte*x extract (2.5 ml/kg l.w.); II group – BD + *Q. corte*x extract (2.5 ml/kg l.w.) + enzyme preparation (5 g/10 kg of feed); III group – BD + enzyme preparation (5 g/10 kg of feed). Enzyme preparation: Glucoamylase (1000 ME) + Xylanase (600 ME). Feeding of the experimental bird was carried out twice a day, feed intake was weighed daily, and *Q. cortex* extract was supplied with drinking water. Every effort was made to minimize the suffering of animals and reduce the number of samples used. Decapitation of birds was carried out under Nembutal ether on the 42^nd^ day.

**Table-1:** Ingredients and nutrient level of basal diets.

Attributes	Control, I, II, III	

	Starter (7-28 days)	Finisher (29-42 days)
Ingredient composition (%)		
Wheat	47	42.0
Barley	2.6	0.3
Corn	7.5	22.0
Soybean meal (46% CP)	25.0	15.0
Sunflower meal (38% CP)	7.0	10.0
Sunflower oil	5.0	5.0
Di-calcium phosphate	1.6	1.4
Mel stern	0.9	1.5
Limestone	0.5	0.3
Salt	0.36	0.2
DL-methionine	0.18	0.16
L-Lysine	0.35	0.17
Vitamin-mineral premix^a^	2.0	2.0
Calculated nutrients Metabolizable energy (kkal/100 g)	296	302.0
Crude protein	22.0	18.7
Methionine+cysteine	0.87	0.79
Lysine	1.35	0.96
Calcium	0.95	1.01
Available phosphorus	0.54	0.48

a Supplied following per kilogram of diet: Vitamin A: 7000 IU; Vitamin D3: 800.0 IU; Vitamin E: 9 IU; Vitamin K3: 1.1 mg; Thiamine: 0.7 mg; Riboflavin: 3.0 mg; Vitamin B6: 1 mg; Vitamin B12: 0.01 mg; Vitamin C: 50 mg; Mn: 23 mg; Fe: 17 mg; Zn: 11 mg; Cu: 2.5 mg; I: 0.4 mg; Se: 0.2 mg

### Preparation of Q. cortex extract

The preparation of the *Q. cortex* extract included 50 g of crushed bark (dosage form) was placed in a heat-resistant dish and 500 ml of hot (70°С) distilled water was added. This mixture was heated in a water bath (30 min), strained and filtered (ashless filter paper “White Ribbon,” d 70 mm APEXLAB).

### Analytical procedures

Blood samples for hematological measures were collected into vacuum tubes with an anticoagulant (EDTA-K3), and for biochemical measures, samples were collected into vacuum tubes with a coagulation activator (thrombin). Hematologic parameters (number and type of leukocytes) were measured using an automatic hematological analyzer URIT-2900 Vet Plus (“URIT Medical Electronic Group Co., Ltd,” China).

The filtered plant extract of *Q. cortex* was analyzed using a gas chromatography–mass spectrometry method with a selective detector GQCMS 2010 Plus (Shimadzu, Japan) on a capillary Column HP-5MS. GCMS Solutions software and GCMS PostRun Analysis were used to interpret the results. CAS, NIST08, Mainlib, Wiley9, and DD2012 Lib spectral libraries were used to identify the compounds. The quantitative presence of individual identified components was estimated by the relative value (%), which relates the peak area to the total area of the extract. 35 compounds of oak bark extract were identified, and substances (10%) showing anti-QS activity on the QS system of the first type were detected [10].

The element composition of tissues and organs were analyzed in the test laboratory of ANO “Centre for Biotic Medicine,” Moscow, Russia (Registration Certificate of ISO 9001: 2000, Number 4017 – 5.04.06). The biosubstrates were ashed in a microwave decomposition system MD-2000 (USA). The content of elements in the resulting ash was measured on a mass spectrometer, Elan 9000, and atomic emission spectrometer, Optima 2000 V (“Perkin Elmer,” USA).

### Statistical analysis

Statistical analysis was performed using IBM “SPSS Statistics Version 20” program, calculating the average value (M), standard deviation (σ), and standard deviation error (m). Results with p≤0.05 were considered significant.

## Results

### Growth performance

According to the results, it was established that an increase in the live weight of broiler chickens in the experimental groups was higher than in the control group. Hence, the difference in age from the 1^st^ to the 4^th^ week between Group I and the control group was 10.7%; between Group II and the control group, it was 16.3% (p≤0.05); and between Group III and the control group, it was 3.1% (Table-2).

**Table-2:** Broiler weight gain (g/head).

Age (days)	Control	Group		

		I^b^	II	III
7-28	1130.3	1251.7^a^	1315.3	1172.1
29-42	936.3	905.6	1095.4	965.3
7-42	2067.0	2157.3	2410.7^a^	2137.4

a p≤0.05 in comparison with the control group;

b Control group - BD

I group - BD+Quercus cortex extract; II group - BD+Quercus cortex extract+enzyme preparation; III group - BD+enzyme preparation

In the second period, in experimental Group I, there was a decrease of 3.3% (p≥0.05), while the increase continued in Group II by 16.9% (p≤0.05). In general, during the experiment in vivarium conditions, the increase in the experimental groups was higher than in the control group by 4.3-16.6%.

Feeding with *Q. cortex* extract promoted an increase in feed intake during the entire experimental period in Group I by 10.6%, against the background of the enzyme-containing diet in Group II by 15.4%, and in Group III by 2.6% (Table-3).

**Table-3:** Feed intake within the experiment (g/head).

Index	Control	Group		

		I	II	III
Starting feed	1348.4	1461.0	1475.6	1387.5
Weight gain feed	1691.5	1901.8	2033.1	1730.4
Total	3039.9	3362.8	3508.7	3117.9

At the same time, feed consumption for a weight gain of 1 kg of live weight decreased by 9.2% in Group II, by 7.8% in Group I, and by 3.7% in Group III.

### Results of biochemical indices

Based on measures of biochemical parameters of blood serum of broiler chickens in the experimental groups, an increase in the endogenous enzyme from the transferase alanine aminotransferase group was registered, which indicates active metabolic processes taking place in liver. In addition, a significant decrease in serum triglycerides was found. The remaining indices were almost at the same level as animals in the control group (Table-4).

**Table-4:** Biochemical blood serum parameters of broiler chickens of Smenq 8 after feeding with *Quercus cortex* extract on the background of enzyme-containing diet (M±m, experiment in vivarium).

Index	Group			

	Control	I	II	III
Total protein (g/l)	32.9±1.69	32.0±1.34	32.2±0.85	32.3±1.13
Albumin (g/l)	17.0±1.09	17.0±0.63	17.2±0.80	16.8±1.22
ALT (units/l)	3.14±0.81	6.1±0.67^a^	5.8±1.16^a^	4.12±0.95
AST (units/l)	235.6±10.55	249.8±7.71	239.3±17.5	237.7±8.25
Total bilirubin (µmol/l)	19.3±0.13	19.4±0.14	19.4±0.14	19.2±0.21
Direct bilirubin (mmol/L)	0.55±0.03	0.53±0.06	0.50±0.03	0.52±0.05
Cholesterol (mmol/L)	5.1±0.24	4.94±0.21	5.3±0.21	5.2±0.23
Triglycerides (mmol/L)	0.21±0.02	0.15±0.04^a^	0.09±0.02^b^	0.18±0.02
Urea (mmol/L)	1.5±0.03	1.46±0.02	1.5±0.07	1.45±0.05
ESR (%)	21.2±2.05	20.4±1.07	21.0±2.7	21.1±1.05
Creatinine (µmol/l)	16.9±1.28	14.64±1.05	19.9±2.6	16.2±1.51
Glucose (mmol/L)	4.91±1.17	4.23±0.4	5.2±0.37	5.1±0.97
Calcium (mmol/L)	3.5±0.15	3.3±0.05	3.3±0.09	3.4±0.08
Phosphorus (µmol/l)	1.8±0.11	1.9±0.12	1.85±0.6	1.8±0.7
Magnesium (mmol/L)	1.35±0.07	1.3±0.04	1.3±0.03	1.4±0.09
Iron (µmol/l)	22.5±1.03	27.4±0.56	28.3±0.94^a^	23.8±0.81

a p≤0.05;

b p≤0.01, in comparison with control group.

ALT: Alanine aminotransferase, AST=Aspartate aminotransferase, ESR=Erythrocyte sedimentation rate

**Figure-1:**
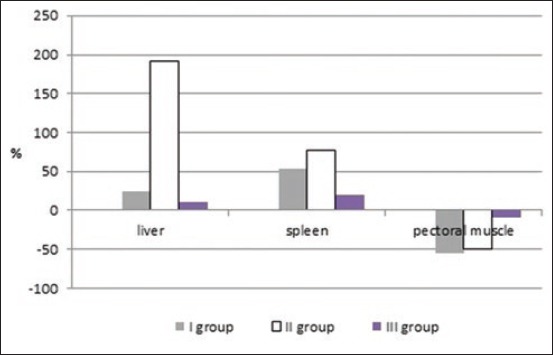
Difference in iron (Fe) content in organs and pectoral muscle of broiler chickens in experimental groups in relation to the control group.

According to the results, there was an increase of iron in the blood of broiler chickens. As can be seen from the chemical analysis of internal organs (Figure-1), the concentration of iron in the liver (as the main depot of this element) of animals of Group II was higher than in animals of the control group and Group I by 2.3-2.9 times (p≤0.05), in spleen by 77.7 (р≤0.05) and 15.4%, and against the background of a decrease in muscle tissue by 50-54.5%. Among the morphological indicators of broiler chicken blood, reduced content of monocytes and granulocytes (Table-5) should be found, especially in Group I.

**Table-5:** Content (10^9^/L) of white blood cells in broilers Smena 8 when feeding with *Quercus cortex* extract against the background of an enzyme-containing diet (M±m, n=20, an experiment in vivarium).

Index	Group			

	Control	I	II	III
Leukocytes	81.7±3.7	78.3±2.3	73.6±4.3	80.8±2.8
Lymphocytes	78.7±3.3	77.4±1.11	69.5±4.2	77.3±2.8
Monocytes	3.5±0.44	2.3±0.16^a^	2.7±0.09	3.2±0.37
Granulocytes	1.7±0.3	0.8±0.13^a^	1.4±0.07	1.6±0.12

a p≤0.05 in comparison with the control group.

## Discussion

The results of the present study on poultry productivity are confirmed by a literature review, particularly on the positive effects of plant extracts as growth promoters [6,14,15]. In addition, a demonstration of antibacterial activity with the use of plant extracts containing tannins in low concentrations [16,17] was previously noted, which helps to reduce the burden on organism microbiota and, as a consequence, this has a favorable effect on the productivity of birds. Previously, researchers [10] have found substances (10%) exhibiting anti-QS activity on a QS system of the first type in *Q. cortex* extract. Due to the presence of tannins in the extract [18], a positive effect on the feeding and growth of animals [14,17,19,20] has been reported, which is confirmed by our study.

The increased feed intake is consistent with the authors stating that low concentrations of tannins in the diet contribute to increased feed intake [21]. This circumstance favorably affected the live weight gain of the experimental birds.

The need for the combined use of *Q. cortex* extract and enzymatic preparation has been shown in the results of previous studies [22-24], which have revealed a manifestation of the tolerance of biologically active substances to gastric content, low pH and bile salts, indicating that tannins can also be used to increase the synergistic effect on the intestinal microbiome. In addition, it is known that xylanase, which is part of an enzymatic preparation against the background of a diet with high wheat content, reduces the pathological effects of *Clostridium perfringens* in broiler chickens [25]. The mechanism of action of glucoamylase and β-glucanase can also be associated with the cleavage and oxidation of glucose to produce gluconic acid and hydrogen peroxide [26], reducing the viscosity of intestinal contents. Accumulating up to a certain level in the organism, hydrogen peroxide inhibits the spread of pathogenic bacteria.

As for the effect of reducing triglycerides in the blood, some authors have observed a decrease in lipid oxidation in the body against a background of increased productivity after adding tannins to the ration of birds [27].

The increase in iron in organs and tissues of experimental birds is probably related to the ability of tannins to deprive iron (one of the mechanisms of positive action) through inhibition of complex forming enzymes in bacteria [28,29]. Since iron is essential for the vital activity of most pathogenic bacteria [30], this can have a positive effect on poultry health; in this case, additional studies are needed.

The decreased content of monocytes and granulocytes is probably explained by the stress of the immune system as a result of activation of the metabolism in the body of bird. Other researchers [31] earlier noted the effect of tannin on the direct modulation of the immune system of chickens as one of the mechanisms of action.

## Conclusion

In the experiment, it was shown for the first time that the inclusion of *Q. cortex* extract in the enzyme-containing diet (anti-quarantine substances) was found to increase the productivity of poultry.

## Authors’ Contributions

GKD and ASU designed the plan of work. NMK, AFR, and BSN conducted the experiment, carried out the laboratory work and analyzed the results. GKD and ASU drafted the manuscript. All authors read and approved the final manuscript.

## References

[1] Diarra M.S, Silversides F.G, Diarrassouba F, Pritchard J, Masson L, Brousseau R, Bonnet C, Delaquis P, Bach S, Skura B, Topp E (2007). Impact of feed supplementation with antimicrobial agents on growth performance of broiler chickens*Clostridium perfringens*and*Enterococcus*counts, and antibiotic resistance phenotypes and distribution of antimicrobial resistance determinants in*Escherichia coli*. Appl. Environ. Microbiol.

[2] Horn N.L, Ruch F, Miller G, Ajuwon K.M, Adeola O (2016). Determination of the adequate dose of garlic diallyl disulfide and diallyl trisulfide for effecting changes in growth performance, total-tract nutrient and energy digestibility, ileal characteristics, and serum immune parameters in broiler chickens. Poultry Sci.

[3] Redondo L.M, Redondo E.A, Delgado F, La Sala L, Pereyra A, Garbaccio S, Fernandez M.M (2013). Control of*Clostridium perfringens*necrotic enteritis by tannins added to the diet. In:Proceedings of the 8^th^International Conference on the Molecular Biology and Pathogenesis of the Clostridia (*Clost Path 8*).

[4] Tosi G, Massi P, Antongiovanni M, Buccioni A, Minieri S, Marenchino L, Mele M (2013). Efficacy test of a hydrolysable tannin extract against necrotic enteritis in challenged broiler chickens. Ital. J. Anim. Sci.

[5] Vondruskova H, Slamova R, Trckova M, Zraly Z, Pavlik I (2010). Alternatives to antibiotic growth promoters in prevention of diarrhoea in weaned piglets:A review. Vet. Med.

[6] Hashemi S.R, Davoodi H (2010). Phytogenies as new class of feed additive in poultry industry. J. Anim. Vet. Adv.

[7] Trufanov O (2016). Phytobiotics in broiler rations. Livest Breeding Rus.

[8] Huyghebaert G, Ducatelle R, Van Immerseel F (2011). An update on alternatives to antimicrobial growth promoters for broilers. Vet. J.

[9] Deryabin D.G, Tolmacheva A.A (2014). Medicinal plants are sources of inhibitors of the Quorum sensing system in bacteria. J. Problems Biol. Med. Pharmac. Chem.

[10] Deryabin D.G, Tolmacheva A.A (2015). Antibacterial and anti-quorum sensing molecular composition derived from*Quercus cortex*(oak bark) extract. Molecules.

[11] Bedford M.R, Cowieson A.J (2012). Exogenous enzymes and their effects on intestinal microbiology. Anim. Feed. Sci. Technol.

[12] Apajalahti J, Kettunen A, Graham H (2004). Characteristics of the gastrointestinal microbial communities with special reference to chickens. World Poult. Sci. J.

[13] Fisinin V.I, Egorov I.A, Lenkova T.N, Okolelova T.M, Ignatova G.V, Shevyakov A.N, Panin I. G, Grechishnikov V. V, Vetrov P. A, Afanasiev V. A, Ponomarenko Yu A (2009). Methodical Instructions on Optimization of Recipes for Mixed Fodders for Agricultural Poultry. Guidelines for the Optimization of Animal Feed Recipes for Poultry.

[14] Hashemi S.R, Davoodi H (2011). Herbal plants and their derivatives as growth and health promoters in animal nutrition. Vet. Res. Comm.

[15] Abreu A.C, McBain A.J, Simões M (2012). Plants as sources of new antimicrobials and resistance-modifying agents. Nat. Prod. Rep.

[16] Simões M, Bennett R.N, Rosa E.A.S (2009). Understanding antimicrobial activities of phytochemicals against multidrug resistant bacteria and biofilms. Nat. Prod. Rep.

[17] Redondo L.M, Chacana P.A, Dominguez J.E, Fernandez M.M.E (2014). Perspectives in the use of tannins as alternative to antimicrobial growth promoter factors in poultry. Front. Microbiol.

[18] Maznev N (2004). Encyclopedia of Medicinal Plants.

[19] Yang C, Chowdhury M.A.K, Huo Y, Gong J (2015). Phytogenic compounds as alternatives to in-feed antibiotics:Potentials and challenges in application. Pathogens.

[20] Schiavone A, Guo K, Tassone S, Gasco L, Hernandez E, Denti R, Zaccarati I (2008). Effects of a natural extract of chestnut wood on digestibility performance traits and nitrogen balance of broiler chicks. Poultry Sci.

[21] Windisch W, Schedle K, Plitzner C, Kroismayr A (2008). Use of phytogenic products as feed additives for swine and poultry. J. Anim. Sci.

[22] Blaiotta G, La Gatta B, Di Capua M, Di Luccia A, Coppola R, Aponte M (2013). Effect of chestnut extract and chestnut fiber on viability of potential probiotic*Lactobacillus*strains under gastrointestinal tract conditions. Food Microbiol.

[23] Kamboh A.A, Zhu W.Y (2014). Individual and combined effects of genistein and hesperidin on immunity and intestinal morphometry in lipopolysacharide-challenged broiler chickens. Poultry Sci.

[24] Mansoori B, Rogiewicz A, Slominski B.A (2015). The effect of canola meal tannins on the intestinal absorption capacity of broilers using a D-xylose test. J. Anim. Physiol. Anim. Nutr.

[25] Liu D, Guo S, Guo Y (2012). Xylanase supplementation to a wheat-based diet alleviated the intestinal mucosal barrier impairment of broiler chickens challenged by*Clostridium perfringens*. Avian Pathol.

[26] Geisen R (1999). Inhibition of food-related pathogenic bacteria by god-transformed*Penicillium nalgiovense*strains. J. Food Prot.

[27] Starčević K, Krstulović L, Brozić D, Maurić M, Stojević Z, Mikulec Ž, Bajić M, Mašek T (2014). Production performance meat composition and oxidative susceptibility in broiler chicken fed with different phenolic compounds. J. Sci. Food Agric.

[28] Hee D.B, McAllister T.A, Yanke J, Cheng K.J, Muir A.D (1993). Effects of condensed tannins on endoglucanase activity and filter paper digestion by*Fibrobacter succinogenes*S85. Appl. Environ. Microbiol.

[29] Mila I, Scalbert A, Expert D (1996). Iron withholding by plant polyphenols and resistance to pathogens and rots. Phytochemistry.

[30] Chung K.T, Lu Z, Chou M.W (1998). Mechanism of inhibition of tannic acid and related compounds on the growth of intestinal bacteria. Food Chem. Toxicol.

[31] Allen H.K, Levine U.Y, Looft T, Bandrick M, Casey T.A (2013). Treatment, promotion, commotion:Antibiotic alternatives in food-producing animals. Trends Microbiol.

